# Sequencing and characterization of mitochondrial genome of *Idaea effusaria* (Lepidoptera: Geometridae)

**DOI:** 10.1080/23802359.2020.1720542

**Published:** 2020-02-03

**Authors:** Jian-Lin Xie

**Affiliations:** Taiyuan University of Science and Technology, Taiyuan, China

**Keywords:** *Idaea effusaria*, complete mitogenome, Geometridae

## Abstract

The *Idaea effusaria* belongs to Geometridae in Lepidoptera. The complete mitogenome of *I. effusaria* was described in this study, which is typically circular duplex molecules and 16,161 bp in length, containing the standard metazoan set of 13 protein-coding genes, 22 transfer RNA genes, 2 ribosomal RNA genes, and an A + T-rich region. The gene order is same with other lepidopterans. Except for *cox1* started with CGA, all other PCGs started with the standard ATN codons. Most of the PCGs terminated with the stop codon TAA, whereas *cox2* has the stop codon CAT. The phylogenetic tree showed that Larentiinae is close to Sterrhinae. The species of Ennominae form a monophyly.

*Idaea effusari*a (Christoph) is a moth of the family Geometridae. Geometridae is one of the most species-rich groups in Lepidoptera, include more than 26,000 described species (Liu et al. 2014). However, mitochondrial genomes of only 13 species in Geometridae were reported. This group is so large and should be studied by more mitochondrial genomes information.

In this study, the samples were collected by light trapping in Taiyuan city of China (37°83′33″N, 112°66′61″E) in July 2019, some of these specimens were immediately frozen in −80 °C on board for mitogenome analysis and others were preserved by spreading wings in the Herbarium of Institute of Plant Protection, Shanxi Academy of Agricultural Sciences, and their numbers is 2019TYKD1706-1710. Total genomic DNA was extracted from tail tip using the Ezup pillar genomic DNA extraction kit (Sangon Biotech, Shanghai, China). The mitogenome was sequenced by Illumina Hiseq 4000. Gene annotation was performed and circularity was checked using the MITOS2 webserver (Bernt et al. 2013, http://mitos.bioinf.uni-leipzig.de/).

The mitochondrial genome of *I. effusaria* has a total length of 16,161 bp (GenBank accession No. MN646772), consisting of 13 PCGs, 22 tRNA, 2 rRNA genes, and an A + T-rich region. The major strand encodes a larger number of genes (9 PCGs and 14 tRNAs) than the minor strand (4 PCGs, 8 tRNAs, and 2 rRNA genes). Gene content and arrangement are highly conserved and typical of Lepidoptera (Wu et al. 2016). The mitogenome is highly biased toward A/T, contains 41.02% T, 41.07% A, 10.70% C, and 7.20% G, which is a feature commonly present in insects (Boore 1999).

All of the protein**-**coding genes have ATN as the start codon except for *cox1*, which starts with CGA. Eleven PCGs have the common stop codon TAA, *cox2* has the stop codon CAT. All tRNAs exhibit typical clover-leaf secondary structure, except for tRNA-Ser(AGN) lacking the DHU arm, which is common in Lepidoptera insects (Garey and Wolstenholme 1989). The 16S rRNA is 1286 bp in length and the 12S rRNA is 720 bp in length. The A + T-rich region is 771 bp long located between 12S rRNA and tRNA-Met and it is longer than other most Lepidoptera insects. There is a motif ATAGA in downstream of 12S rRNA followed by an 17 bp Poly-T stretch.

The phylogenetic position of *I. effusaria* was inferred using sequences of the 13 PCGs of 14 species. Thirteen of them belong to Geometridae and a species from Pyraloidea (which is used as outgroup) (Figure 1). The sequences were aligned with MAFFT v7.2 software (Katoh and Standley 2013), the evolutionary analyses were conducted with RAxML v8.2.10 (Stamatakis 2014) on the CIPRES Science Gateway (Miller et al. 2010). The result showed that *I. effusaria* and *Operophtera brumata* are clustered into a clade, Larentiinae is close to Sterrhinae. Other moths belong to Ennominae and form a monophyly. The result is consistent with morphological classification.

**Figure 1.**
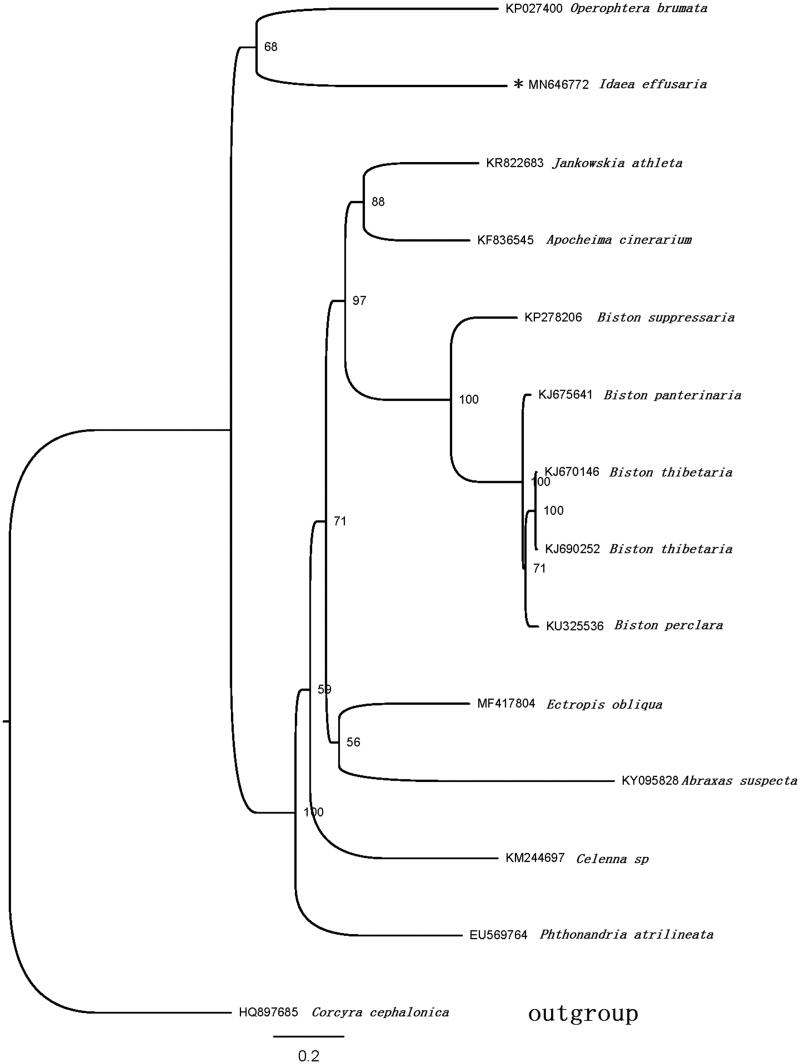
Maximum-likelihood tree of evolutionary relationships *I. effusaria* based on the complete mitogenomes of 14 Lepidopteran moths.

## Nucleotide sequence accession number

The complete mitochondrial genome sequence of *I. effusaria* was deposited in the GenBank under the accession number MN646772.
